# In vitro and in vivo evaluation of a new phytotherapic blend to treat acute externa otitis in dogs

**DOI:** 10.1111/jvp.13000

**Published:** 2021-07-13

**Authors:** Cristina Vercelli, Mario Pasquetti, Giovanni Giovannetti, Sara Visioni, Giovanni Re, Mario Giorgi, Graziana Gambino, Andrea Peano

**Affiliations:** ^1^ Department of Veterinary Science of Turin University of Turin Turin Italy; ^2^ Nutrigen LG Distribuzione S.r.l Prato Italy; ^3^ Department of Veterinary Science of Pisa University of Pisa Pisa Italy

**Keywords:** dog, essential oils, otitis, phytotherapic, topical administration

## Abstract

Canine otitis externa is frequently encountered in veterinary practice, caused by primary factors with bacteria and yeast overgrowth acting as secondary and perpetuating factors. The pharmacological support includes anti‐inflammatory, antimicrobials, and antimycotic drugs, but therapeutic failure and antimicrobial resistance are leading to alternative strategies based on phytotherapic products. This study aimed to evaluate an essential oil blend (Otogen^®^) to treat otitis externa in dogs. The experimental design was divided in: (a) an *in vitro* approach, based on the European Normative UNI EN 1275:2006, to assess the efficacy of the product against the most frequently isolated microorganisms during otitis externa. (b) an *in vivo* part, 12 owned dogs presenting with acute otitis externa were enrolled. A significant growth reduction (>99.9%) of *Malassezia pachydermatis* and *Candida albicans* after 15 min of contact and *Pseudomonas aeruginosa* after 1 h of incubation was recorded. For *Staphylococcus pseudintermedius*, 50% of growth reduction were appreciated after 15 min. Results obtained *in vivo* after 7 days of blend administration, noted a significant improvement of all the considered parameters (most important were head shaking, erythema, and scraping). The results obtained may support the usefulness of the tested phytotherapic blend to manage acute otitis externa in dogs.

## 1 INTRODUCTION

Canine otitis externa (OE) is an inflammatory pathology commonly reported in veterinary clinical practice. During the early stages, inflammation results in erythema of the pinnae, external meatus, and lining of the external canal. Subsequently, there can be a wide range of clinical signs, such as head shaking, ear scratching, ceruminous, or purulent discharge, excoriations due to self‐trauma, malodor, swelling, and pain. In recurrent or chronic cases, clinical signs may progress to proliferative changes leading to stenosis of external ear canal, and ultimately to occlusion (Guarda et al., 2013). In such a scenario bacteria or yeasts act as perpetuating causes of the inflammatory process, since they are not responsible for the initiation of the OE but permit to continue once established and can lead to pathology chronicity (Bajwa, 2019; Guaguère & Prèlaud, 2005). *Staphylococcus* and *Pseudomonas* are bacteria able to produce biofilm that acts as protection and can lead to therapeutic failure (Bajwa, 2019), while among yeasts, the species by far more prevalent is *Malassezia pachydermatis* (Guillot & Bond, 2020).

In clinical practice, most cases of acute OE are managed using polyvalent topical ear products that include a glucocorticoid (used to control mild acute inflammation), and antimicrobial agents used to treat concurrent infections (Nuttall, 2016). Various detergents, such as ethylenediaminetetraacetic acid (TrizEDTA) and chlorhexidine, are largely used to clean the ear canal, remove debris, and excessive cerumen production and to disrupt biofilm (Guardabassi et al., 2009; Pye, 2018). Antibiotics, especially fluoroquinolones, aminoglycosides, and polymyxins, should be used only after the identification of etiological agents and only after ascertaining the integrity of the tympanic membrane (Ghibaudo et al., 2015). Treatments against *M. pachydermatis* include antifungal drugs like azole derivatives (thiabendazole, clotrimazole, miconazole, and itraconazole), nystatin, and terbinafine. The main causes of therapeutic failure are incorrect patient medication management, and the lack of identification of primary conditions (Nuttall, 2016) and progressing to otitis media force to switch to systemic therapy (Six et al., 2000).

Antimicrobial resistance (AMR) is a global threat for humans and animals, with a significant public health risk due to AMR transmission between these two populations considering the side‐by‐side style of life among humans and pets (WHO, 2015). Antimicrobial stewardships have been recently proposed in veterinary medicine and should be emphasized for frequently diagnosed pathologies (Vercelli et al., 2021), such as canine otitis externa (Chan et al., 2020). Resistance has been demonstrated for bacteria that can alter target sites, increased drug outflow, and enhance enzymatic degradation (Wright, 2005), and other mechanisms have been recently described also for M. pachydermatis (Angileri et al., 2018; Peano et al., 2017; Peano et al., 2020). Considering all the aforementioned factors, it does not surprise the increasing interest in alternative therapies. Medicinal plant‐derived products represent today between 25% and 50% of pharmaceutical products (Gerwick, 2013; Gupta & Birdi, 2017).

The antimicrobial properties of medicinal plant extracts come from the large variety of secondary metabolites. These are intermediate or final products of plant metabolism, not fundamental for life plant processes, playing a defensive role toward bacteria, fungi, protozoa, and viruses (Gorlenko et al., 2020). Secondary metabolites include quinines, alkaloids, lecithins, polypeptides, flavones, flavonoids, coumarin, terpenoids, essential oils, and tannins (Chandra et al., 2017). The applications of plant‐derived products are increasing, and mainly direct to treat parasitic disease and skin pathologies (López et al., 2019; Tresch et al., 2019). Some studies have been performed in the last years to investigate the efficacy of essential oils (EOs) in otitis externa in dogs. Still, the majority are *in vitro*, and only one is *in vivo* (Nardoni et al., 2017; Sim et al., 2019; Sim, Khazandi, Pi, et al., 2019; Song et al., 2020).

The present study aimed to investigate the efficacy of a new phytotherapic blend containing essential oils with a double approach: first an *in vitro* evaluation on the most frequently diagnosticated microorganisms in case of canine otitis externa was performed. Then an *in vivo* trial was organized, evaluating the efficacy the same phytotherapic blend in dogs presenting with spontaneous acute otitis externa.

## 2 MATERIALS AND METHODS

### 2.1 Blend composition

The commercially available Otogen^®^ formulation was provided by the producing company (Nutrigen s.r.l., Prato, Italy). The commercial product includes essential oils of *Melaleuca alternifolia* (also named tea tree oil – TTO), *Thymus serpillum*, *Salvia officinalis*, *Eucalyptus officinalis*, *Rosmarinus officinalis*, *Macadamia alternifolia*, *Lavandula officinalis*, and *Heliantus annus*, as active compounds, and helianthus seed oil (HSO), isopropile miristate, isopropile adipate and a mixture of triglycerides as excipients. The different properties of the EOs are summarized in Table 1. The acidity of the blend was stated at 0.22% ± 0.02. The measurement was performed according to the European Regulatories 2568/1991 and 2016/1227 by a certified laboratory (Appendix S1).

**TABLE 1:** properties of essential oils present in Otogen blend (Bozin et al., 2007; Carson et al., 2006; Rasooli & Mirmostafa, 2000; Woronuk et al., 2011)

Activity	Natural component							
	MalalEuca alternifolia	Thymus serpillum	SAlvia officinalis	Eucaliptus officinalis	Rosmarinus officinalis	Anternifolia macadamia	Lavandula officinalis	Heliantus annus
Germicidal	X							
Antimicrobial	X							
Antibacterial		X	X	X	X		X	
Antifungal	X	X			X			
Antiseptic	X	X		X			X	
Antioxidant			X					X
Hydrating						X		
Emollient						X		
Skin‐regenerating			X			X		X
Anti‐inflammatory	X							

### 2.2 In vitro assays

The *in vitro* efficacy of Otogen^®^ blend was assessed following the method of the European Normative UNI EN 1275 for the evaluation of fungicidal or yeasticidal activity of chemical disinfectants and antiseptics (Anon., 2005), with some modifications. The method consists in evaluating the number of living microorganisms after the contact with antiseptics at different time points.

The organisms tested included one clinical strain of *M. pachydermatis*, *Pseudomonas aeruginosa*, and *Sthaphylococcus pseudintermedius* and an ATCC strain of *Candida albicans* (ATCC strain 90028).

The microorganism inoculums (Test Suspension ‐ TS) were prepared by picking some bacterial or yeast colonies and suspending them into sterile tubes with HSO. The TS was vortexed for 5 min to obtain a preparation as homogeneous as possible. The use of HSO instead of distilled water recommended in the Normative (Anon., 2005), was necessary since the phytotherapic blend under test is a mix of oils. The possible effects of HSO on microorganisms were assessed in preliminary experiments, and it was established that HSO does not affect microorganism viability (data not shown).

As regards the inoculum size, we could not reach that indicated in the Normative (1–5 × 10^7^ colony‐forming units [CFUs]/ml), due to the high viscosity of HSO. Thus, in our experiments, the inoculum sizes were: 4.5 × 10^6^ [CFUs]/ml (*M. pachydermatis*); 1.9 × 10^5^ CFUs/ml (*C. albicans*); 1.2 × 10^6^ CFUs/ml (*S. pseudintermedius*); 3.1 × 10^5^ CFUs/ml (*P. aeruginosa*).

For each test, 1 ml of TS was added in a tube with 9 ml of the blend (final concentration of the blend 90%, blend test suspension ‐ BTS 90), and in a tube with 5 ml of the blend plus 4 ml of HSO (final concentration of the blend 50% ‐ BTS 50). After 5, 15, and 60 min of contact, 50 µl of the different suspensions were seeded in neutralizing media: Sabouraud dextrose agar with Tween 80 30 g/L and lecithin 3 g/L was used for *M. pachydermatis* and *C. albicans*; Mueller Hinton agar with Tween 80 30 g/L and lecithin 3 g/L was used for bacteria. After incubation at 37℃ for 48–72 h and 24 h for yeasts and bacteria, respectively, CFUs in each Petri dish were counted and the reduction compared with the TS was calculated. According to the Normative (Anon., 2005) a product may be considered "effective" when causing at least a 4 decimal log reduction of the germ number (i.e., a reduction equal to 99,99%) after a 15‐min contact time.

### 2.3 In vivo evaluation

To perform the *in vivo* evaluation of the phytotherapic blend, we enrolled owned dogs presenting with clinical symptoms of acute otitis externa. Owners signed an informed consent before the beginning of the trial. Dogs could be of any breed, weight, sex, or neuter status, provided that they were at least 8‐week‐old. Exclusion criteria consisted in the administration of systemic or topical drugs within the last 2 months. It was hypothesized to withdraw patients during the study for the following reasons: adverse events, administration of concomitant therapy, owner incompliance, or any other documented reason. Each ear was considered separately, as a single case: this is because each ear can have different anatomy and a unique microenvironment. Table 2 reports the description of the 12 dogs enrolled in the study.

**TABLE 2:** Descriptive data of the dogs enrolled in the study

*n*°	Breed	Age (Year)	Sex	Neutered	Weight (kg)
1	Labrador retriever	1.5	M	No	28
2	Golden retriever	12	F	Yes	43
3	Mix breed	5	M	Yes	25
4	Cavalier King Charles Spaniel	5	M	No	10
5	Newfoundland	14	F	Yes	50
6	Bernese Mountain Dog	5	F	No	45
7	German Shepherd	1	F	No	25
8	Weimaraner	6	F	Yes	25
9	Maremma shepherd	6	M	Yes	31
10	German Shepherd	12	F	No	30
11	Maremma shepherd	5	F	Yes	32
12	Mixed breed	4	F	Yes	23

At the first visit, a complete physical examination was performed by a veterinarian. All the information regarding the dog, past and recent anamnesis, general and objective examinations, signs of otitis and findings at the otoscopic evaluation were recorded. At the same time, a sample was collected for a cytological exam. Otogen^®^ was administered once a day for seven consecutive days, using cotton soaked in the product. At the end of the treatment, a complete physical examination, including otological exam, and a new cytological exam were performed.

### 2.4 Ear examination

Both ears of each dog were examined. The investigator scored the severity of nine clinical signs – for each ear ‐ of otitis externa divided into two main groups:

- Parameters investigated by clinical history and physical examination: head tilt, shaking/discomfort, pain, pruritus, and bad smell.
- Parameters investigated by otoscopic examination (following the method OTIS3 by Nuttall & Bensignor, 2014, with slight modifications): ‐ erythema, edema/swelling, exudate, and quantification of earwax.

Scores for each parameter were given on a severity scale of 0–3 (0 = none; 1 = mild; 2 = moderate; 3 = marked). The sum of the scores yielded the total score for each ear (maximum score 27).

The presence of mites, ulcers, and foreign bodies was also recorded.

### 2.5 Cytological exam

Cerumen samples were collected using a swab prior to the first and after the last administration. Slides were prepared by rolling the swabs on their surface. They were stained by the Wright's technique (Merchant, 2005) and observed microscopically for the presence of yeasts (*Malassezia*) and bacteria (cocci and rods).

Following a semiquantitative criterium (Merchant, 2005), the presence of microorganisms was evaluated, as follows:

‒ *Malassezia* (observation at 40X, mean count considering 10 fields):
• Mean count ≤2: normal.
• Mean count 3–4: intermediate growth.
• Mean count ≥5: overgrowth.
‒ Bacteria (observation at 100X, mean count considering 10 fields):
• Mean count ≤5: normal.
• Mean count >5 ≤ 24: intermediate growth.
• Mean count ≥25: overgrowth.

The presence of inflammatory cells (neutrophils and macrophages), eventually with bacteria within them, was also recorded as an evidence of actual infection.

### 2.6 Effectiveness evaluation criteria

At the control visit, a complete cure (ear "recovered") was defined as a return to normal of all parameters (sum of scores = 0).

Secondary criteria were also considered:

- an improvement between 80% and 100% of the initial severity score was considered as "strong improvement";
- between 60% and 80% "clear improvement";
- between 40% and 60% "improvement";
- between 0% and 40%: the dog was considered as "steady";
- if total score of the final visit was higher than that of the first examination the condition was considered as "worsened".

According to cytological findings, pathogens were considered normalized in case the score went to 0. Other evaluations were possible (improved, unmodified, worsened) basing on the comparison of the score pre‐ and post‐treatment.

## 3 RESULTS

### 3.1 In vitro study

#### 3.1.1 Contact assays

**FIGURE 1:**
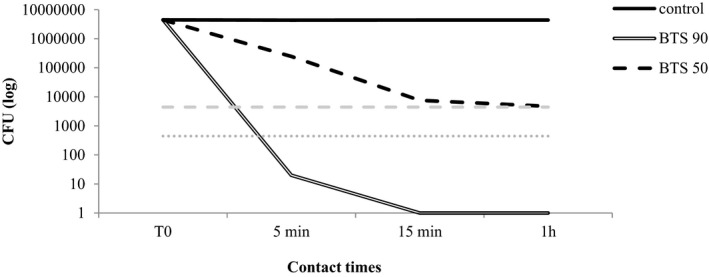
Activity of two concentrations of the phytotherapic blend (90%, BTS 90; 50%, BTS 50) against *M. pachydermatis* after different contact times. The dotted lines indicate the threshold value below which the reduction from the starting inoculum is 99.9% (dashed gray line) and 99.99% (dotted gray line)

**FIGURE 2:**
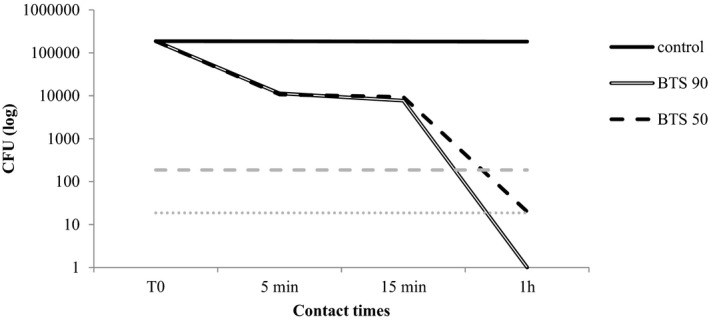
Activity of two concentrations of the phytotherapic blend (90%, BTS 90; 50%, BTS 50) against *C. albicans* after different contact times. The dotted lines indicate the threshold value below which the reduction from the starting inoculum is 99.9% (dashed gray line) and 99.99% (dotted gray line)

**FIGURE 3:**
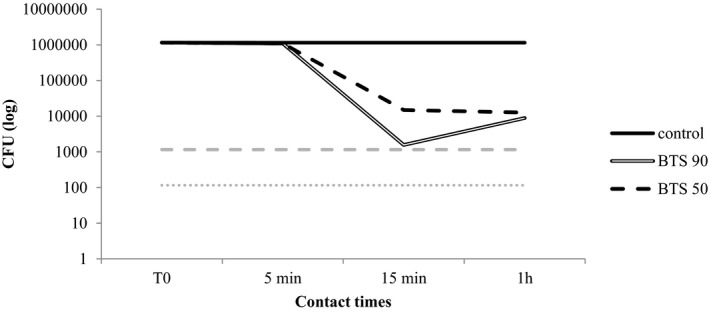
Activity of two concentrations of the phytotherapic blend (90%, BTS 90; 50%, BTS 50) against *Staphylococcus pseudointermedius* after different contact times. The dotted lines indicate the threshold value below which the reduction from the starting inoculum is 99.9% (dashed gray line) and 99.99% (dotted gray line)

**FIGURE 4:**
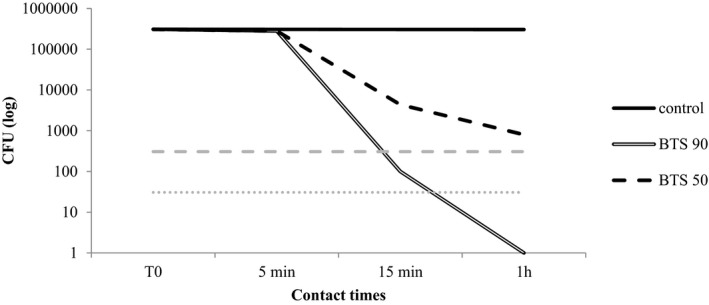
Activity of two concentrations of the phytotherapic blend (90%, BTS 90; 50%, and BTS 50) against *Pseudomonas aeruginosa* after different contact times. The dotted lines indicate the threshold value below which the reduction from the starting inoculum is 99.9% (dashed gray line) and 99.99% (dotted gray line)

The results obtained are presented in Table 3 and Figures 1, 2, 3, 4. As regards the BTS 90, the highest efficacy was shown against *M. pachydermatis* (99,99% reduction already after 5 min of incubation) followed by *C. albicans* and *P. aeruginosa* (99,99% reduction after 1 h of incubation). For *S. pseudintermedius*, the activity was good, though the reduction did not reach 99.99%. The activity of the blend diluted at 50% was generally inferior, but anyway around 99% in many cases after 15 min or 1 h of contact (Table 3).

**TABLE 3:** CFU (colony‐forming unit) reduction after contact with the phytotherapic blend at different contact times (nc = non‐countable)

Time of contact											
			5 min			15 min			1 h		
Microorganism	CFUs/ml in the starting inoculum	Blend concentration under test	CFU	% of growth compared with starting inoculum	% of germ reduction	CFU	% of growth compared with starting inoculum	% of germ reduction	CFU	% of growth compared with starting inoculum	% of germ reduction
*M. pachydermatis*	4.5 × 10^6^	90%	20	0.0004	99.9996	0	0.0000	100.0000	0	0.0000	100.0000
		50%	246800	5.5303	94.4697	7600	0.1703	99.8297	4740	0.1062	99.8938
*C. albicans*	1.9 × 10^5^	90%	11400	6.0881	93.9119	7720	4.1228	95.8772	0	0.0000	100.0000
		50%	10760	5.7463	94.2537	9400	5.0200	94.9800	20	0.0107	99.9893
*S. pseudintermedius*	1.2 × 10^6^	90%	nc	–	–	1560	0.1344	99.8656	8860	0.7631	99.2369
		50%	nc	–	–	14960	1.2885	98.7115	12700	1.0939	98.9061
*P. aeruginosa*	3.1 × 10^5^	90%	nc	–	–	100	0.0325	99.9675	0	0.0000	100.0000
		50%	nc	–	–	4360	1.4179	98.5821	800	0.2602	99.7398

### 3.2 In vivo study

**FIGURE 5:**
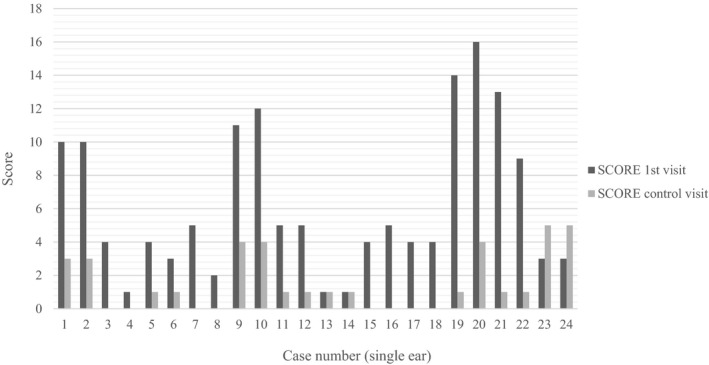
Scores regarding clinical and otoscopic signs of otitis before and after treatment considering each case (case = single ear) (*N* = 24)

Figure 5 presents the scores indicating the severity of clinical signs of otitis, considering single ears, before and after treatment. The figure reports, for each ear, the sum of scores regarding all the parameters considered (results for the individual parameters are available in Appendix S2). Table 4 shows data about the assessment of treatment efficacy. Main outcome (complete recovery) is considered together with secondary criteria. Mites, ulcers, and foreign bodies were not found either during the first visit or during the control visit.

**TABLE 4:** Outcome of treatment

	*n*	%	*n* (cumulative)	% (cumulative)
Cure	8	33.3	8	33.3
Strong improvement	5	20.8	13	54.2
Clear improvement	7	29.2	20	83.3
Steady	2	8.3	22	
Worsened	2	8.3	24	
Total	24			

No adverse effects were recorded and none of the cases was withdrawn.

### 3.3 Cytological exam

Inflammatory cells were not seen in any of the cases. As regards microorganisms, the presence of cocci and *Malassezia* was noted in 18 and 12 cases, respectively. Rods were not present.

The number of microorganisms decreased after the treatment with Otogen: 11 cases were normalized and 3 were improved for cocci. Only 3 cases were unchanged and 1 was worsened out of 18. *Malessezia* decreased till the normalization in 9 cases, unchanged in 2 cases and worsed only in 1 case out of 12 (details are available in Appendix S3).

## 4 DISCUSSION

The present study permitted to evaluate the efficacy of a new phytotherapic blend during canine acute otitis externa, using a dual experimental approach. The results obtained by the *in vitro* assays are suggestive of high efficacy against *M. pachydermatis*. The BTS 90 was able to induce a significant (>99,99%) decrease of yeast growth after 5 min of incubation. The present results are in line with those obtained by other investigators, though these latter employed a different susceptibility test (Hammer et al., 2003): TTO MICs for all test fungi ranged from 0.004% to 0.25% and minimum fungicidal concentrations (MFCs) ranged from <0.03% to 8.0%. These data demonstrate that TTO has both inhibitory and fungicidal activity. Even if the BTS 50 was not able to reach the cutoff value, it was able to induce an appreciable reduction of the concentration of *Malassezia*. These data should be considered positively in the perspective of clinical application: an incomplete administration (i.e., a small amount administered or product accidental removal) might be sufficient to limit the yeast proliferation.

Contact assays with *C. albicans* and *P. aeruginosa* demonstrated a significant inhibition after 1 h of incubation in the presence of BTS 90. The BTS 50 was able to limit microorganism's growth without reaching the cutoff value.

The results obtained with *S. pseudintermedius* demonstrated the incomplete efficacy of BST 90 and 50, though the reduction percentage approximated the significant value of 99.99%. Interestingly, the number of CFUs was higher after 1 h contact than 15 min. This phenomenon may be due to a bacteriostatic or slightly bactericidal action, that may hesitate in a partial inhibition of the pathogen.

Among the different EOs contained in the phytotherapic blend, *Melaleuca alternifolia* and *Salvia officinalis* have been considered as potent antibacterial agents and confirmed efficacy obtained in the present study (Tresch et al., 2019). Essential oils could thus be included in the treatment, as an alternative therapeutic option, alone or in combination with allotherapic approach (Ebani et al., 2017).

The essential oils are known to possess antimicrobial activity (Mickienė et al., 2011): particularly TTO is the most effective contrasting the growth of streptococci, enterococci, staphylococci, as well as having an antifungal effect against yeasts and dermatophytes (Carson et al., 2006). Some studies reported that TTO could have a sensitizing potential if used pure or in high concentrations, or in case of oxidation of its components terpinene‐4‐ol and α‐terpinene (Groot & Schmidt, 2016). In the present study, the concentration of TTO in the blend did not cause any allergic reaction, indicating a good biocompatibility of the entire formulation. It seems also to possess a potent bactericidal activity toward multiresistant bacteria such as methicillin‐resistant *Staphylococcus aureus* (MRSA), *K. pneumoniae* resistant to carbapenem, *Acinetobacter baumannii* e *Pseudomonas aeruginosa* (Olivia et al., 2018). *Salvia officinalis*, *Eucalyptus officinalis*, and *Lavandula officinalis* are also able to control the growth of cocci and bacilli involved in the development of otitis externa. *Salvia officinalis* also shows an antifungal effect that can counteract the growth of *Candida* spp. (Oliveira, Vilela, et al., 2019) while lavender has a possible application against parasite infestations, including those caused by mites (Cavanagh & Wilkinson, 2002). The control of the inflammatory process can be mediated by the extracts of the Eucalyptus which, thanks to its high content of 1 – 8 cineole, is able to suppress the production of pro‐inflammatory cytokines (Juergens et al., 1998). To control pain, the essential oil of *Rosmarinus officinalis* is able to achieve an antinociceptive effect, and demonstrates antibacterial effect against *Streptococcus* spp., *Staphylococcus* spp., *Pseudomonas aeruginosa*, and *Candida albicans*, all involved in the development of the ear inflammatory process (Oliveira et al., 2019).

A limit of the present study may be the inoculum size employed in *in vitro* tests. The number of CFUs was indeed different from organism to organism, and in none of the experiments, we could reach the size indicated in the standard procedure followed (Anon., 2005). Due to the fact that we tested the final commercial product, we had to suspend the germs in oil, which did not allow to prepare a homogeneous and reproducible inoculum. Using "classical" broth‐dilution methods (whose results are expressed as MIC values) would have allowed us to overcome this technical problem and obtain a more accurate and reproducible evaluation of the activity of the blend components. We decided to use contact tests instead, because we think that they are more predictive of *in vivo* outcome of topical treatment. Contact tests allow to take into account the main factors which influence the efficacy of antimicrobial topical products, namely the product formulation effects and the duration of contact (Russel & McDonnel,^2000^). This in turn allows simulating what happens when the final marketed formulation is applied on skin or in the ears. Lloyd and Lamport (1999) demonstrated that formulation is an important factor affecting the antimicrobial efficacy of topical products (chlorhexidine in that case). In marketed products, other principles (i.e., surfactants) can interact with the active principles. Contact tests have been employed in another study on the activity of topical formulations employed to treat dermatitis and otitis in dogs (Nebbia et al., 2008).

Our study was performed to have a preliminary idea on the activity of the phytotherapic blend against the most representative microorganisms involved in canine OE. Therefore, only one isolate for each species was tested *in vitro*. A point of strength is that 3 out of 4 microorganisms were of clinical provenance, the results obtained in the *in vitro* experiments are easily comparable to real cases.

Considering the data obtained by the *in vivo* part it was possible to appreciate that several breeds presenting with signs of otitis externa were recruited, without concomitant pathologies. All the clinical signs of otitis externa were improved after a daily administration in most dogs, and owner's compliance was high and mainly because they appreciated the pleasant scent, able to immediately reduce the unpleasant odor frequently occurring in course of otitis. The amelioration of typical signs of otitis, such as head shaking and pruritus, is an important point in the cure approach of this pathology, where auto‐traumatism can worsen the clinical situation. Considering the cytological parameters, the macroscopic improvement is positively correlated with the decrease of the presence of cocci and *Malassezia*. A global consideration of all the above results can lead to the conclusion that the phytotherapic blend is able to induce a cleaning of the canal that permits to active substances to reestablish ear homeostasis, reducing the inflammation process and enhance global clinical conditions of the patient. According to the authors' knowledge, few papers investigated the role of essential oils to treat otitis externa. None evaluated the efficacy of a complex blend like that used in the present study (Nardoni et al., 2017; Sim, Khazandi, Chan, et al., 2019).

The results obtained in the present study should be worthy of attention and can be considered a starting point for further investigations on the efficacy of plant‐derived compounds. The tested bacteria demonstrated in the last years increasing antimicrobial resistance phenomena. The total or partial inhibition obtained by the phytotherapic blend used in the present study suggests a good efficacy of EOs against these pathogens.

All the above considerations come from selected subjects that do not present chronic otitis, and it is not possible to extend these considerations to complicated otitis externa. The Authors' advice is to consider Otogen to clean and regulate the wetting of the ear environment in case of complicated otitis externa but further studies are needed to collect information specifically addressed to this condition.

## 5 CONCLUSION

The phytotherapic blend tested solution could be regarded as valuable support to cure acute otitis externa in dogs.

The improvement shown for both the clinical and cytological examinations in most of the enrolled cases leads to the hypothesis that the product has good effectiveness in restoring and maintaining homeostasis of the auricle environment.

## CONFLICT OF INTEREST

The authors declare that the study received financial support by Nutrigen LG Distribuzione S.r.l., necessary to purchase materials. No other conflicts of interest have to be declared in publishing this work.

## AUTHOR CONTRIBUTIONS

CV and AP designed the experimental procedures, coordinate the different experimental phases and wrote the draft of the paper, and CV performed the visits, MP and SV performed the analysis, and enrolled patients, GG^1^, GG^2^, GR, and MG supervised all the procedures, revised the results, checked the draft of the paper.

## Supporting information

Appendix S1

Appendix S2

Appendix S3

## Data Availability

All relevant data are within the paper and supporting information files.
