# Supplementary material for: In vitro and in vivo evaluation of a new phytotherapic blend to treat acute externa otitis in dogs
Source: J Vet Pharmacol Ther. 2021 Jul 13;44(6):910–8. doi: 10.1111/jvp.13000 (PMC9290716; doi:10.1111/jvp.13000)
Supplement: Supplementary file 1 — Appendix S1 [file JVP-44-910-s002.pdf]

pH Labs

RAPPORTO DI PROVA

N° 21-LA17079

Numero di identificazione del campione: 21-LA17079

(C) Descrizione del campione: Otogen 200ml - Lotto OC70

(C) Campionamento effettuato da: Cliente (§)

Ritiro effettuato da: Corriere

(C) Richiedente: LG DISTRIBUZIONE S.R.L.
Via Valentini 7
PRATO 59100 PO

Data arrivo campione: 13/04/2021

(§) Il laboratorio declina ogni responsabilità sul campionamento. I risultati si riferiscono al campione così come ricevuto.
(C) Informazioni fornite dal Cliente/Terzi

ESITO D'ESAME

Prova / Metodo — Prove Chimiche	Risultato	Inc	u.m.	LOQ	LOD	Limiti	Rec.	u.o.	Note	Data Inizio	Data Fine
Acidità (come acido oleico)<br>*Reg CEE 2568/1991 11/07/1991 GU CEE L248 05/09/1991 All II + Reg UE 2016/1227 27/07/2016 GU UE L202 28/07/2016 All I*	0.22	±0.02	%	0.05	0.02			0_A		14/04	19/04

Legenda:
Inc (Incertezza); u.m. (unità di misura); LOQ (limite di quantificazione); LOD (limite di determinazione); Rec. (recupero); u.o. (unità operativa); 0_A (prova eseguita presso u.o. di Barberino Tavarnelle - FI, via Sangallo); 0_B (prova eseguita presso u.o. di Barberino Tavarnelle - FI, via Bramante); 0_D (prova eseguita presso u.o. di Tito Scalo); II (lab. mobili); III (analisi in esterna); LE.# (prova eseguita in subappalto c/o laboratorio terzo. PH Srl è responsabile verso il cliente per il lavoro subappaltato, eccetto il caso in cui il cliente specifichi quale laboratorio debba essere impiegato);

NOTE

- Per le prove chimiche, i valori di incertezza estesa sono riferiti ad un intervallo di confidenza del 95%. Fattore di copertura k=2. Dove non indicato diversamente, il limite di determinazione (LOD) risulta uguale a 3/10LOQ.
- Il laboratorio utilizza il punto come separatore delle cifre decimali.
- Nel caso sia presente una Dichiarazione di Conformità, il Laboratorio adotta come regola decisionale il confronto diretto del risultato con il limite applicato senza tenere conto dell'incertezza di misura.
- I risultati riportati sono riferiti al solo campione sottoposto a prova.
- I campioni alimentari ed i campioni non deteriorabili sottoposti ad analisi sono conservati per 30 giorni dalla data di arrivo del campione. Campioni di acque, compost e di altre matrici deteriorabili sono conservati fino all'emissione del Rapporto di Prova.
- pH srl è iscritta al numero 013 dell'elenco regionale dei laboratori che effettuano analisi nell'ambito delle procedure di autocontrollo delle industrie alimentari (L.R. Toscana n°9 09/03/2006).

Il presente rapporto di prova non può essere riprodotto in forma parziale salvo l'approvazione scritta del Laboratorio. Il rapporto di prova originale viene fornito in formato digitale e firmato con sistema di firma digitale certificata dal responsabile autorizzato (file 21-LA17079.p7m). Eventuali copie stampate del suddetto documento digitale originale non hanno validità legale.

P.H. s.r.l. Società unipersonale soggetta al controllo e al coordinamento di TÜV SÜD AG e-mail: info@phsrl.it web: www.tuv.it/ph
- Sede legale e Laboratorio Alimenti: Via Sangallo, 29 50028 Barberino Tavarnelle (FI) telefono: +39 055 80961 fax: +39 055 8071099
- Uffici e Laboratorio Ambiente: Via Bramante, 10/12 50028 Barberino Tavarnelle (FI) telefono: +39 055 80677 fax: +39 055 8067850
- Laboratorio Ambiente: Z.I. Tito Scalo 85050 Tito (PZ) telefono: +39 0971 485795 fax: +39 0971 485795

pH Labs

N° 21-LA17079

Li, 19/04/2021

(Nota: la data sopra riportata rappresenta la data di redazione del presente rapporto di prova. La data di emissione del rapporto di prova corrisponde con la data di apposizione della firma digitale)

per il Responsabile di Laboratorio
dr.ssa Elena Ciofi

ORDINE DEI CHIMICI · TOSCANA
DOTT. ELENA CIOFI
CHIMICO
A-2046

--- Fine del Rapporto di Prova ---

Il presente rapporto di prova non può essere riprodotto in forma parziale salvo l'approvazione scritta del Laboratorio. Il rapporto di prova originale viene fornito in formato digitale e firmato con sistema di firma digitale certificata dal responsabile autorizzato (file 21-LA17079.p7m). Eventuali copie stampate del suddetto documento digitale originale non hanno validità legale.

P.H. s.r.l. Società unipersonale soggetta al controllo e al coordinamento di TÜV SÜD AG e-mail: info@phsrl.it web: www.tuv.it/ph
- Sede legale e Laboratorio Alimenti: Via Sangallo, 29 50028 Barberino Tavarnelle (FI) telefono: +39 055 80961 fax: +39 055 8071099
- Uffici e Laboratorio Ambiente: Via Bramante, 10/12 50028 Barberino Tavarnelle (FI) telefono: +39 055 80677 fax: +39 055 8067850
- Laboratorio Ambiente: Z.I. Tito Scalo 85050 Tito (PZ) telefono: +39 0971 485795 fax: +39 0971 485795
